# Phytochemical Composition, Antioxidant and Antimicrobial Activity of the Balkan Endemic *Micromeria frivaldszkyana* (Degen) Velen. (Lamiaceae)

**DOI:** 10.3390/plants10040710

**Published:** 2021-04-07

**Authors:** Tsvetelina Mladenova, Plamen Stoyanov, Petko Denev, Stela Dimitrova, Mariana Katsarova, Desislava Teneva, Krasimir Todorov, Anelia Bivolarska

**Affiliations:** 1 Department of Botany and Methods of Biology Teaching, Faculty of Biology, University of Plovdiv “Paisii Hilendarski”, 24 Tzar Assen Street, 4000 Plovdiv, Bulgaria; cmladenova@uni-plovdiv.bg (T.M.); pstoyanov@uni-plovdiv.bg (P.S.); ktodorov@uni-plovdiv.bg (K.T.); 2 Department of Bioorganic Chemistry, Faculty of Pharmacy, Medical University of Plovdiv, 15A Vasil Aprilov Boulevard, 4000 Plovdiv, Bulgaria; Stela.Dimitrova@mu-plovdiv.bg (S.D.); kacarovam@abv.bg (M.K.); 3 Laboratory of Biologically Active Substances, Institute of Organic Chemistry with Centre of Phytochemistry, Bulgarian Academy of Sciences, 139 Ruski Boulevard, 4000 Plovdiv, Bulgaria; petkodenev@yahoo.com (P.D.); desi_gerinska@yahoo.com (D.T.); 4 Department of Medical Biochemistry, Faculty of Pharmacy, Medical University of Plovdiv, 15A Vasil Aprilov Boulevard, 4000 Plovdiv, Bulgaria

**Keywords:** *Micromeria frivaldszkyana*, polyphenols, antioxidant activity, antimicrobial activity

## Abstract

The current study investigates the content of sugars, organic acids, phenolic acids and flavonoids, as well as antioxidant and antimicrobial activity of Balkan-endemic *Micromeria frivaldszkyana*. Glucose was the most abundant sugar in the plant (2.77%), followed by fructose (1.18%) and galactose (0.82%). Eight organic acids were detected with quinic acid being in the highest content—556.3 mg/100 g DW. From the individual phenolic acids, rosmarinic acid was found in the most significant amounts (2040.1 ± 1.97 mg/100 g) and hesperidin was the major representative of flavonoids with content 131.2 ± 5.6 mg/100 g DW. The antioxidant activity of the plant was studied by six methods: 2,2′-diphenylpicrylhydrazyl (DPPH)—286.4 ± 10.43 mM TE/g, 2,2′azinobis (3)-ethylbenzthiazoline-6-sulfonic acid (ABTS)—358.4 ± 10.4 mM TE/g, ferric reducing antioxidant power (FRAP)—388.0 ± 32.4 mM TE/g, cupric reducing antioxidant capacity (CUPRAC)—905.6 ± 19.2 mM TE/g, Oxygen Radical Absorbance Capacity (ORAC)—3250.5 ± 208.1 µmol TE/g and Hydroxyl Radical Averting Capacity (HORAC)—306.1 ± 23.5 µmol GAE/g. In vitro antimicrobial activity against nine microorganism was evaluated but the extract displayed antimicrobial activity only against *Listeria monocytogenes* ATCC 19111 with inhibition zone diameter 9 mm and minimal inhibitory concentration (MIC) 10 mg/mL.

## 1. Introduction

In recent years, there has been a growing trend in the global population, as about 80% of people worldwide rely on the use of herbal medicines and supplements for their primary health care needs [1,2]. This growing demand for herbal medicines encourages new research and drug developments [2]. In fact, many active ingredients of new drugs are derived from medicinal plants that have been shown to be extremely important in supporting the discovery and development of drugs [3]. It is therefore necessary to carry out active research on plants in order to identify potential candidates as safer and more effective agents in the future.

The Lamiaceae family includes numerous popular and lesser-known plants with pronounced therapeutic effects. This large botanical family is taxonomically divided into several subfamilies, one of the largest being Nepetoideae, which includes species with pronounced antioxidant properties [4]. The genus *Micromeria* Bentham also belonging to this subfamily is represented by about 70 species, 21 of which are distributed in Europe [5]. A number of molecular and morphological studies have shown a close relationship between the genera *Micromeria* (section Pseudomelissa) and *Clinopodium* [6,7] and refer the representatives of the genus *Micromeria* to the genus *Clinopodium* [7]. Several *Micromeria* species have been reported as antirheumatic, antiseptic, abortifacient, CNS stimulant, and tonic and several polyphenolic compounds have been identified in them [8]. Polyphenols reduce inflammatory mediators, leukocyte migration and stabilize endothelial cells, thus being involved in the pathogenesis of vascular disorders [9]. Traditionally, members of the genus *Micromeria* are used against heart diseases, respiratory diseases (asthma), headaches, colds, wounds, skin infections, as well as for insecticidal, herbicidal and culinary purposes [10,11,12].

In Bulgaria, the genus *Micromeria* is represented by four species: *Micromeria juliana* (L.) Bentham ex Reichenb., *Micromeria cristata* (Hampe) Griseb., *Micromeria dalmatica* Bentham ssp. bulgarica (Velen.) and *Micromeria frivaldszkyana* (Degen) Velen. The species *Micromeria frivaldszkyana* is a Bulgarian endemic [13], included in Appendix 3 of the Biological Diversity Act [14] and the Red Data Book of the Republic of Bulgaria [15] under the category endangered. The species was assessed according to the criteria of IUCN [16] under the category almost endangered (EN) and appears in the Red List of higher plants in Bulgaria [17] under the category endangered (EN). The species is a perennial herbaceous plant, distributed mainly in the rocky regions of Central and Eastern Stara Planina in Bulgaria.

Medicinal plants contain different substances (i.e., flavonoids, alkaloids, tannins, and terpenoids) that reveal antioxidant and antimicrobial effects. Due to their antimicrobial properties, extracts from various medicinal plants have been widely used for treatment of different diseases. Further, many studies investigated the possible application of plant extracts as natural preservatives. There are several studies on the antiinflammatory and antimicrobial effects of *Micromeria* against some pathogens [8,10,18]. Ali-Shtayeh et al. reported that the antimicrobial activity of different *Micromeria* species’ extracts could be attributed to plant’s phenolic compounds [19]. However, antimicrobial properties of *Micromeria frivaldszkyana* are barely studied. Phytochemical studies of rare and endemic plants with specific habitats are of scientific interest and thus the assessment of the biological characteristics, distribution and natural resources of the species *Micromeria* is important for their conservation and sustainable use [20]. Previous studies report only the 2,2′-diphenylpicrylhydrazyl (DPPH) radical scavenging activity of *M. frivaldszkyana* and TLC analysis of the flavonoid profiles of four *Micromeria* species distributed in Bulgaria [12]. More detailed information on phytometabolites and biological activity of *M. frivaldszkyana* is still missing. Therefore, the aim of the current study was to investigate the content and composition of sugars, organic acids and major polyphenols of the Balkan-endemic *Micromeria frivaldszkyana*, and to evaluate its antioxidant and antimicrobial effects.

## 2. Results and Discussion

### 2.1. Sugar and Organic Acid Content and Composition

The content and composition of sugars in *M. frivaldszkyana* aerial parts are presented in Table 1. As it is evident, glucose was the prevalent sugar in the investigated plant with content of 2.77%, followed by fructose (1.18%) and galactose (0.82%). In addition, the disaccharides sucrose and melibiose were detected in the plant. Total amount of sugars was calculated to be 5499 mg/100 g.

The content and composition of organic acids in *M. frivaldszkyana* aerial parts are presented in Table 2. In total, eight organic acids were detected with quinic acid being in the highest content—556.3 mg/100 g DW. Citric acid was also present in considerable amounts—341.5 mg/100 g DW.

Studies on the chemical composition of the Bulgarian-endemic plant *M. frivaldszkyana* are very scarce. However, there are a few studies on related species from the same family [21], as well as on the essential oil of the plant [22]. To our knowledge, we report for the first time the content and composition of sugars and organic acids from *M. frivaldszkyana* aerial parts. The metabolism of organic acids is essential at the cellular level for several biochemical pathways, including energy production and the formation of precursors for amino acid biosynthesis, in the plant’s adaptation to the environment. The total content of organic acids in plant tissues is higher than in other organisms. The composition of the organic acids that accumulate varies depending on the type and age of the plant and the type of tissue. The high level of organic acids in plant tissues is most likely due to their important role as photosynthetic intermediates. However, organic acids have a potential role as metabolically active solutes for the osmotic adjustment and the balance of cation excess [23].

### 2.2. Polyphenol Content and Composition

Table 3 presents data on the content of major phenolic components in *M. frivaldszkyana* aerial parts. Unlike other plants, *M. frivaldszkyana* shows very high content of rosmarinic acid, and from the individual phenolic compounds, it was found in the most significant amounts (2040.1 ± 15.76 mg/100 g). In plants, rosmarinic acid is considered a cumulative protective compound, while in humans it has many biological activities, including antiviral, antibacterial, antioxidant, antimutagenic and anti-inflammatory [24]. Many in vitro and in vivo studies have reported the antiinflammatory effects of rosmarinic acid in inflammatory diseases [25]. In their study, Al-Hamwi et al. also observed the presence of chlorogenic acid (1.123 mg/g) and ferulic acid (0.05 mg/g) in ethanolic extracts of *M. fruticosa* [26]. Other studies indicate the presence of chlorogenic acid (7.409 mg/g) and ferulic acid (0.597 mg/g) in ethanolic extract of *M. barbata* [27]. The presence of quercetin, rutin, naringin, chlorogenic acid and rosmarinic acid has also been found in methanolic extracts of *M. frivaldszkyana* by thin layer chromatography [28]. In a qualitative study of four species of *Micromeria* apigenin, luteolin and their derivatives were found in acetone extract of *M. cristata* and *M. juliana*, whereas chlorogenic acid was detected in methanol extracts of *M. dalmatica* and *M. frivaldszkyana* [12]. Chlorogenic and rosmarinic acids were detected in ethanol extracts of three *Micromeria* species of—*M. croatica, M. juliana* and *M. thymifolia* by Vladimir-Knežević et al. [29]. Phenolics are among the most common secondary plant metabolites, being synthesized to protect plants against various environmental conditions such as drought, UV radiation, pests or physical injury [30]. Several flavonoids (epicatechin, hesperidin, quercetin, kaempherol and apigenin) in *M. frivaldszkyana*, and hesperidin was the major representative with content of 131.2 ± 5.6 mg/100 g DW. Flavonoids, as one the largest groups of plant phenolics, are found in many lower and higher plant species [31]. Many studies reveal that flavonoids have valuable medicinal properties, which determines the great research interest in this group of biologically active substances.

### 2.3. Antioxidant Activity Determined by Different Assays

There are many papers attempting to rank the antioxidant properties of plant materials using different methods, and for better assessment of antioxidant properties several methods should be used [32]. Therefore, to evaluate antioxidant activities of investigated ethanol extracts, their abilities to scavenge DPPH and 2,2′azinobis (3)-ethylbenzthiazoline-6-sulfonic acid (ABTS) radicals, their power to reduce ferric (FRAP) and cupric (CUPRAC) ions, their ability to neutralize peroxyl radicals (Oxygen Radical Absorbance Capacity—ORAC), as well as the complexing ability of an antioxidant under Fenton reaction conditions (Hydroxyl Radical Averting Capacity—HORAC) were investigated (Table 4). It is evident from the results that *M. frivaldszkyana* aerial parts are a rich source of phenolic compounds (9004 ± 129.8), including flavonoids (594.2 ± 3.6). This renders high antioxidant activity, measured via different assays. The antioxidant activity of the plant was as follows: DPPH—286.4 ± 10.43 mM TE/g, ABTS—358.4 ± 10.4 mM TE/g, FRAP—388.0 ± 32.4 mM TE/g, CUPRAC—905.6 ± 19.2 mM TE/g, ORAC—3250.5 ± 208.1 µmol TE/g and HORAC—306.1 ± 23.5 µmol GAE/g. Interestingly, ORAC value of *M. frivaldszkyana* is higher than many other Bulgarian medicinal plants [33]. Many polyphenols exhibit antioxidant properties, actively participating in the redox processes of the cell. They can neutralize free radicals by releasing an electron or a hydrogen atom. Polyphenols inhibit the formation of free radicals by reducing the rate of oxidation by inhibiting the formation or deactivation of ROS [34]. Flavonoids and phenolic acids are considered to be the most important factors for the antioxidant activity of medicinal plants [12].

### 2.4. Antimicrobial Activity

Antimicrobial tests of *M. frivaldszkyana* against nine microorganisms were carried out by disc diffusion assay and determination of minimal inhibitory concentration (Table 5). Antibiotics penicillin and nystatin were used as positive controls and inhibition zone above 7 mm was considered as a positive result. The extract of *M. frivaldszkyana* demonstrated no antimicrobial activity against most of the tested microorganisms. Only the Gram-positive bacteria—*Listeria monocytogenes* ATCC 19111 was sensitive to the tested *M. frivaldszkyana* extract (inhibition zone (IZ), 9 mm and minimal inhibitory concentration (MIC), 10 mg/mL). To the best of our knowledge, there is no published data for the antimicrobial activity of *M. frivaldszkyana* extract against *Listeria monocytogenes*, which is one of the most virulent foodborne microorganisms, also acting as a saprophyte or a pathogen, depending on the environment. Nystatin exhibited strong inhibitory effect against all fungi, but penicillin just to *Staphylococcus aureus* ATCC 25923 and *Listeria monocytogenes* ATCC 19111. There are only a few data about the antimicrobial effect of *Micromeria* species. The leaf extract has been used to treat fever, chest pain, skin infections and gastrointestinal symptoms like stomach pain [35]. *Micromeria* spp. (*M. graeca* autc. Non L., *M. fruticosa* (L.) Druce) are well known to contain essential oil and flavonoids [36]. Brahmi et al. observed antibacterial activity of the *Micromeria* extract against four pathogenic bacteria, *E. coli* ATCC 25922, *P. aeruginosa* ATCC 9027, *S. aureus* ATCC 6538, and *S. aureus* 100459, the extracts were inactive against all test-microorganisms (MIC ˃ 2000 μg/mL) [18]. Ali-Shtayeh et al. reported that the freeze-dried water extract of *M. nervosa* (Desf.) Benth. (origin, Palestine) indicated lowest activity [19]. According to Brahmi et al. nonpolar extracts from plants have been shown more effective antibacterial properties than polar extracts [18]. Despite the high polyphenol and flavonoid content (26,570.5 ± 291.3 mg/100 g DW and 1564 mg/100 g DW, respectively) of the used extract, we detected antimicrobial activity against only one foodborne pathogen. This is most probably due to the phenolic profile of the used extracts, since it is known that the antimicrobial properties of different classes’ polyphenolic compounds varies significantly [37].

## 3. Materials and Methods

### 3.1. Chemicals

All analytical standards, Trolox, fluorescein disodium salt, AAPH and Folin-Ciocalteu’s reagent, were obtained from Sigma-Aldrich (Steinheim, Germany). All other solvents used were of analytical grade and purchased from local distributors.

### 3.2. Plant Material

Aerial parts of *M. frivaldszkyana* were collected August 2019 on the territory of Bulgarka Nature Park, Stara Planina (middle) floristic region. The species was determined according to Delipavlov and Cheshmedzhiev [38]. The material was air dried and kept in paper bags before analysis. To clarify the chorology of the studied species, the Flora of Europe was used [5]. Herbarium material from *M. frivaldszkyana* was deposited under No. 062648 in the herbarium of the Agricultural University—Plovdiv (SOA).

### 3.3. Extraction of Polyphenols

The extraction of phenolic compounds was performed according to Denev et al. [39]. Before extraction and analysis, samples were milled in a laboratory mill to fine powder. Briefly, approximately 0.5 g of the *M. frivaldszkyana* powder were weighted accurately, transferred to an extraction tube and mixed with 40 mL 60% ethanol, *v*/*v*. Samples were extracted at room temperature on a magnetic stirrer for 1 h. After that, extracts were filtered and centrifuged (6000× *g*, 20 min) and supernatants were further used for antioxidant activity determination and analyses of phenolic compounds.

### 3.4. Extraction of Sugars and Organic Acids

The extraction of carbohydrates and organic acids was performed according to Denev et al. with some modifications [39]. Briefly, one gram of the *M. frivaldszkyana* powder was weighted accurately and extracted for 1 h, at 30 °C with 30 mL distilled water and shaking on a thermostatic water bath (NUVE, Ankara, Turkey). After that, the samples were filtered and centrifuged (6000× *g*, 20 min) and the supernatants were used for HPLC analysis of sugars and organic acids [39].

### 3.5. High Performance Liquid Chromatography (HPLC) Analysis of Sugars

HPLC determination of sugars was performed according to Denev et al. on HPLC system Agilent 1220 (Agilent Technology, Santa Clara, CA, USA), with a binary pump and Refractive Index Detector (Agilent Technology, USA) [39]. The column was Zorbax Carbohydrate (150 × 4.6 mm, 5 μm, Agilent), connected to a guard column Zorbax Reliance Cartridge (Agilent), and as eluent was used 80% acetonitrile in water at a flow rate of 1.0 mL/min and temperature 25 °C. Results were calculated with standards curves for each analytical standard (fructose, glucose, galactose, sorbitol, rhamnose, xylose, arabinose, sucrose and melibiose) and expressed as mg/100 g dry weight (DW) [39].

### 3.6. HPLC Determination of Organic Acids

HPLC determination of organic acids was performed according to Denev et al. on HPLC system Agilent 1220 (Agilent Technology, USA), with binary pump and UV-Vis detector (Agilent Technology, USA) [39]. Organic acid separation was performed on Agilent TC-C18 column (250 × 4.6 mm, 5 μm) at 25 °C and the eluate was monitored at 210 nm. The mobile phase was 25 mM phosphate (K_2_HPO_4_/H_3_PO_4_) buffer (pH 2.4), flowing at 1.0 mL/min. Results were calculated with standards curves for each analytical standard (quinic acid, malic acid, ascorbic acid, citric acid, α-ketoglutaric acid, succinic acid, oxalic acid, formic acid, shikimic acid and tartaric acid) and expressed as mg/100 g DW.

### 3.7. HPLC Determination of Phenolic Compounds

The obtained extracts were filtered through 0.25 μm microfilter and injected into the HPLC system. Qualitative and quantitative determinations of phenolic acids and flavonoids, were performed by using Waters 1525 Binary Pump HPLC systems (Waters, Milford, MA, USA), equipped with Waters 2484 dual Absorbance Detector (Waters, Milford, MA, USA) and Supelco Discovery HS C18 column (5 µm, 25 cm × 4.6 mm), at 25 °C, operated under control of Breeze 3.30 software. The sample was injected at 20 µL volume and gradient elution at flow rate of 1.0 mL/min was performed by using the following setup:
**Time, min****1% Acetic Acid, (%)****Methanol, (%)**090.010.03678.022.03770.030.04760.040.05854.046.05940.060.07120.080.07290.010.07590.010.0

Gallic acid, protocatehuic acid, (+)-catechin, vanillic acid, syringic acid, (−)-epicatechin, p-coumaric acid, salicylic acid and hesperidin were detected at 280 nm, whereas chlorogenic acid, caffeic acid, ferulic acid, rutin, rosmarinic acid, quercetin and kaempherol were detected at 360 nm. Results were calculated with standards curves for each analytical standard and expressed as mg/100 g DW.

### 3.8. Total Polyphenol Compounds Analysis

The total polyphenol content was determined spectrophotometrically with the Folin–Ciocalteu’s reagent according to the method of Singleton and Rossi with some modifications [40]. Briefly, 0.1 mL of the extracts were mixed with 3.1 mL deionized water and 0.2 mL of Folin–Ciocalteu phenol reagents. After 3 min, 0.6 mL of 20% sodium carbonate was added to the mixture. Samples were incubated for 5 min at 50 °C and their absorbance was measured at 765 nm. Gallic acid was employed as a calibration standard and the results were expressed as mg gallic acid equivalents (GAE) per 100 g DW.

### 3.9. Total Flavonoid Content Analysis

The total flavonoid content was determined with AlCl_3_ reagent according to Chang et al. [41]. The calibration curve was constructed with quercetin dihydrate (10–200 mg/L). The results are expressed as mg quercetin equivalents (QE) per 100 g DW.

### 3.10. Antioxidant Activities

Oxygen Radical Absorbance Capacity (ORAC) assay was measured according to the method of Ou et al. [42], with some modifications, described by Denev et al. [43]. The antioxidant activity was expressed in micromole trolox equivalents (µmol TE) per gram DW.

The Hydroxyl Radical Averting Capacity (HORAC) assay measures the metal-chelating activity of antioxidants in the conditions of Fenton-like reactions employing a Co(II) complex and hence the protecting ability against formation of hydroxyl radical [44]. The results are expressed in micromole gallic acid equivalents (µmol GAE) per gram DW.

The DPPH assay uses the stable radical 2,2′-diphenylpicrylhydrazyl (DPPH) as a reagent according to the method of Ivanov et al. [45]. The antioxidant activity was expressed as mM Trolox equivalents (TE) per g dry weight (DW).

ABTS (2,2′azinobis (3)-ethylbenzthiazoline-6-sulfonic acid) assay described by Thaipong et al. [46] was used after some modifications according to Ivanov et al. [45].

Ferric reducing antioxidant power (FRAP) assay was performed according to method, described by Benzie and Strain [47] slightly modified by Ivanov et al. [45].

The Cupric reducing antioxidant capacity (CUPRAC) assay was performed according to Apak et al. [48] with some modifications Ivanov et al. [45].

The antioxidant activity of DPPH, ABTS, FRAP, and CUPRAC was expressed as mM (TE)/g DW by using calibration curve, build in range of 0.05–0.5 mM Trolox dissolved in methanol.

### 3.11. Antimicrobial Activity

Extracts for antimicrobial activity determination were obtained by the following procedure: Two grams of the *M. frivaldszkyana* powder were weighted accurately and extracted for 15 min, at 90 °C with 100 mL distilled water in thermostatic water bath. After that, the samples were filtered and centrifuged (6000× *g*, 20 min) and the supernatants were frozen and freeze-dried in Alpha 1–4 LDplus laboratory freeze dryer to powders. The obtained freeze-dried extracts had total polyphenol content of 26,570.5 ± 291.3 mg/100 g DW and total flavonoid content of 1564 mg/100 g DW.

Three Gram-positive bacteria (*Bacillus cereus* ATCC 11778; *Staphylococcus aureus* ATCC 25923; *Listeria monocytogenes* ATCC 19111), two Gram-negative bacteria (*Proteus vulgaris* ATCC 6380; *Escherichia coli* ATCC 8739), three fungi (*Penicillium chrysogenum* ATCC 28089; *Rhizopus arrhizus* ATCC 11145; *Aspergillus niger* ATCC 1015) and the yest *Saccharomyces cerevisiae* were used in the experiments.

Determination of the antimicrobial activity was performed according to Jirovetz et al. [49]. Test-microorganism (concentration ~1 × 10^7^ cfu/cm^3^) suspension was spread on the Luria–Bertani medium with glucose (LBG) agar (LB Broth, Miller–Novagen, Merck, Germany) in plates. Agar disc-diffusion method using 6 mm paper discs, containing 6 μL of the test extract at a desired concentration. After 24–48 h of sample incubation at 37 °C for bacteria and 30 °C for yeast and fungi, the diameter of the inhibition zone (IZ) was measured in millimetres.

Agar serial tube dilution is the most commonly used method to determine the minimal inhibitory concentration (MIC). Experiments were conducted with undiluted and diluted (10- and 100-fold) plant extract in order to determine MICs. Paper discs soaked in distilled water were used as controls. The results were expressed as diameters of the clear zones around the paper discs, in millimeters, after 24–48 h of incubation at optimal temperature for the growth of the corresponding test-microorganism [49]. The MIC assays determine the lowest concentration of an antimicrobial agent that prevents visible growth of a microorganism. The experiments were performed in quadruplicate.

## 4. Conclusions

The antioxidant antimicrobial activity of the rare endemic species *M. frivaldszkyana* (Degen) Velen. was studied for the first time. The amounts of total flavonoids, polyphenols, phenolic acids, organic acids and sugars in the plant were analyzed, as well. The study shows that *M. frivaldszkyana* is a good source of natural antioxidants and particularly rosmarinic acid, and it would be of interest of cultivating this rare endemic plant due to its limited area and inaccessible distribution. In vivo work is needed to utilize and assess the antioxidant and antimicrobial properties of this rare endemic.

## Figures and Tables

**Table 1:** Sugars content and composition of *M. frivaldszkyana* aerial parts.

Fructose, mg/100 g	Glucose, mg/100 g	Galactose, mg/100 g	Sucrose, mg/100 g	Melibiose, mg/100 g	Total, mg/100 g
1184.3 ± 45.1	2776.2 ± 38.6	816.2 ± 28.9	513.3 ± 21.8	206.9 ± 12.8	5499.0

Results are expressed as mean value ± SD.

**Table 2:** Organic acids content and composition of *M. frivaldszkyana* aerial parts.

Quinic Acid, mg/100 g	Malic Acid, mg/100 g	Ascorbic Acid, mg/100 g	Citric Acid, mg/100 g	α-Ketoglutaric Acid, mg/100 g	Succinic Acid, mg/100 g	Oxalic Acid, mg/100 g	Tartaric Acid, mg/100 g
556.3 ± 22.4	91.0 ± 4.8	83.6 ± 5.9	341.5 ± 21.9	36.4 ± 3.0	125.9 ± 8.1	13.1 ± 1.1	45.0 ± 2.9

Results are expressed as mean value ± SD.

**Table 3:** Content of the major phenolic acids and flavonoids in *M. frivaldszkyana* aerial parts.

	Representative	Content, mg/100 g
Phenolic acids	Chlorogenic acid	11.0 ± 0.6
	Ferulic acid	traces
	Rosmarinic acid	2040.1 ± 15.76
	Protocatehuic acid	52.1 ± 4.1
	Vanillic acid	404.8 ± 21.5
	Caffeic acid	178.0 ± 9.2
	Syringic acid	20.5 ± 1.3
	Salicylic acid	75.2 ± 4.1
Flavonoids	(−)-Epicatechin	87.6 ± 3.2
	Hesperidin	131.2 ± 5.6
	Quercetin	7.5 ± 0.3
	Kaempherol	26.4 ± 1.2
	Apigenin	0.96 ± 0.08

Results are expressed as mean value ± SD.

**Table 4:** Total polyphenol content, total flavonoid content and antioxidant activity of *M. frivaldszkyana* aerial parts determined by different assays.

DPPH, mM TE/g	ABTS, mM TE/g	FRAP, mM TE/g	CUPRAC, mM TE/g	ORAC, µmol TE/g	HORAC, µmol GAE/g	Total Polyphenols, mg/100 g	Total Flavonoids, mg/100 g
286.4 ± 10.4	358.4 ± 10.4	388.0 ± 32.4	905.6 ± 19.2	3250.5 ± 208.1	306.1± 23.5	9004 ± 129.8	594.2 ± 3.6

**Table 5:** Antimicrobial activity of *M. frivaldszkyana* (Degen) Velen. extract.

Microorganism	Viable Cell Count of Microorganism in Nutrient Medium, ×10^7^ cfu/cm^3^	*M. frivaldszkyana* Extract 10 mg/mL	Penicillin, 10 mg/mL	Nystatin, 20 mg/mL
		Inhibition Zones, mm		
*Bacillus cereus* ATCC 11778	1.6	-	-	N/A
*Penicillium chrysogenum* ATCC 28089	4.1	-	N/A	18
*Rhizopus arrhizus* ATCC 11145	1.2	-	N/A	26
*Aspergillus niger* ATCC 1015	1.7	-	N/A	30
*Saccharomyces cerevisiae*	1.0	-	N/A	28
*Staphylococcus aureus* ATCC 25923	1.0	-	25	N/A
*Proteus vulgaris* ATCC 6380	7.0	-	-	N/A
*Escherichia coli* ATCC 8739	1.0	-	-	N/A
*Listeria monocytogenes* ATCC 19111	5.0	9 (MIC, 10 mg/mL)	15	N/A

Legend: “-”—no inhibition; N/A—not applied; d well = 6 mm.

## Data Availability

Not applicable.
